# Pediatric Posterior Reversible Encephalopathy Syndrome: A Review With Emphasis on Neuroimaging Characteristics

**DOI:** 10.7759/cureus.51216

**Published:** 2023-12-28

**Authors:** Yahea Alzahrani

**Affiliations:** 1 Department of Internal Medicine, Taif University, Taif, SAU

**Keywords:** clinical presentation, hypertension, ct, neuroimaging, mri, pediatrics, pres, posterior reversible encephalopathy syndrome (pres)

## Abstract

Posterior reversible encephalopathy syndrome (PRES) is a neurological disorder characterized by the sudden onset of seizures, headaches, and visual disturbances. Its exact cause is unknown, but several triggers and associated conditions are identified, including high blood pressure, kidney dysfunction, and various medications. Magnetic resonance imaging (MRI) plays a crucial role in diagnosis due to its high sensitivity and specificity for detecting characteristic features. Pediatric PRES exhibit age-dependent differences in triggers, radiological findings, and clinical course. The lesions typically involve the posterior cortical and subcortical white matter, but atypical locations and features are also observed. While generally reversible with appropriate treatment, PRES carries a risk of permanent neurological damage.

Despite increasing cases, the current literature on pediatric PRES remains limited. This review highlights the need for further research to understand the mechanisms, delineate distinct clinical and radiological features, and develop precise diagnostic and management strategies for pediatric patients.

## Introduction and background

Posterior reversible encephalopathy syndrome (PRES), initially described in 15 adult patients by Hinchey et al. in 1996 [1], also referred to as reversible posterior leukoencephalopathy syndrome, reversible occipital-parietal encephalopathy, and reversible posterior cerebral edema syndrome [2], is a neurological disorder distinguished by a constellation of clinical and radiological findings and characterized by the abrupt onset of neurological symptoms, including seizures, headache, and visual disturbances [3].

The precise pathophysiological mechanisms underlying PRES remain incompletely understood. However, several hypotheses have been proposed, implicating blood-brain barrier (BBB) disruption and subsequent fluid extravasation into the interstitium, leading to cerebral parenchymal edema [4-6]. PRES is triggered by specific conditions and associated with a range of medical conditions and underlying factors. These include kidney dysfunction, autoimmune diseases, blood disorders such as sickle cell anemia, and various types of cancer. PRES is often associated with high blood pressure [1-4]. However, it can also occur in people with normal or mildly elevated blood pressure [7]. Other factors that can trigger PRES include organ transplantation, certain medications, blood transfusions, and HIV infection [6-8].

Neuroimaging, particularly magnetic resonance imaging (MRI), is a crucial diagnostic tool for PRES due to its high sensitivity and specificity for detecting characteristic features [3]. This widespread availability has heightened awareness among physicians and radiologists. The underlying triggers for PRES can vary based on age, and the radiological findings and clinical course may exhibit age-dependent disparities. These differences can be attributed to several critical factors, including the pediatric brain's susceptibility to toxins, unique blood flow regulation mechanisms, and distinct control of blood vessel constriction and dilation [1-12]. Typically, the PRES is characterized by reversible vasogenic edema, predominantly affecting the posterior subcortical white matter, particularly in the parieto-occipital regions [13]. However, atypical MRI features of PRES are usually identiﬁed including location in the frontal lobes, basal ganglia, corpus callosum, cerebellum, and brainstem; gadolinium enhancement; the presence of hemorrhagic changes; and restricted diffusion [13-16]. While PRES is generally considered reversible with timely and appropriate treatment, there is still a risk of permanent neurological damage and even death in some cases. The mortality rate for PRES can be as high as 16% [17,18].

Despite a rise in pediatric PRES cases, the current literature still needs to be expanded to capture its diverse clinical and radiologic presentations. Additionally, its delayed presentation compared to adults presents a unique diagnostic challenge. This necessitates further comprehensive research to elucidate the underlying mechanisms and delineate its distinct clinical and radiological features. This review aimed to elucidate and expand current knowledge of pediatric PRES. By enhancing the understanding of both radiologists/neuroradiologists and pediatricians, this review seeks to facilitate the development of more precise diagnostic and optimized management strategies for pediatric patients affected by this condition.

## Review

Epidemiology and predisposing factors

PRES is a rare but potentially serious neurological disorder affecting children. Its estimated incidence in the general pediatric population is approximately 0.04% [19], increasing to 0.7% in children with cancer and 0.4% in those admitted to the pediatric intensive care unit (PICU) [20]. There are significantly more female patients than male patients [2].

Notably, among children receiving autologous hematopoietic cell transplantation, the incidence of PRES rises to 5.2% for those experiencing complications [21]. Nationwide surveys suggest a shift in the age of presentation towards adolescence, with a mean age of 12.5 years. This contrasts with earlier studies on pediatric oncology patients with PRES, which reported a lower mean age range of seven to nine years and a potential female predisposition [22].

Underlying medical conditions significantly influence the risk of PRES in children. Common associations include renal diseases, systemic lupus erythematosus (SLE), sickle cell disease, and bone marrow or solid organ transplantation [1,4]. Particularly, renal insufficiency of various etiologies stands as the leading trigger, followed by hematologic diseases and their associated treatments, including cytotoxic agents and corticosteroids used for malignancy or immunosuppression [9,19,23]. Furthermore, specific chemotherapeutic agents and total body irradiation have been identified as contributing factors to PRES development in children.

PRES was mainly linked to immunosuppressive therapy and sudden blood pressure spikes [1,24,25]. Similar to adults, primary/secondary hypertension and kidney diseases are the significant risk factors for PRES in children and adolescents [14,19]. Additionally, immunosuppressants used after transplants (e.g., tacrolimus, cyclosporine A) significantly increase the risk. PRES has also been associated with cancers and chemotherapy drugs like vincristine and methotrexate. Autoimmune disorders and treatments (e.g., rituximab) are also significant risk factors [14,24-27].

Blood transfusions are significantly associated with PRES, as reported in several studies [19,26-29]. Other contributing factors include sepsis, electrolyte imbalances, Guillain-Barré syndrome, Crohn's disease, dialysis, and trauma [14,19,26-30].

Pathophysiology of PRES

**Figure 1:**
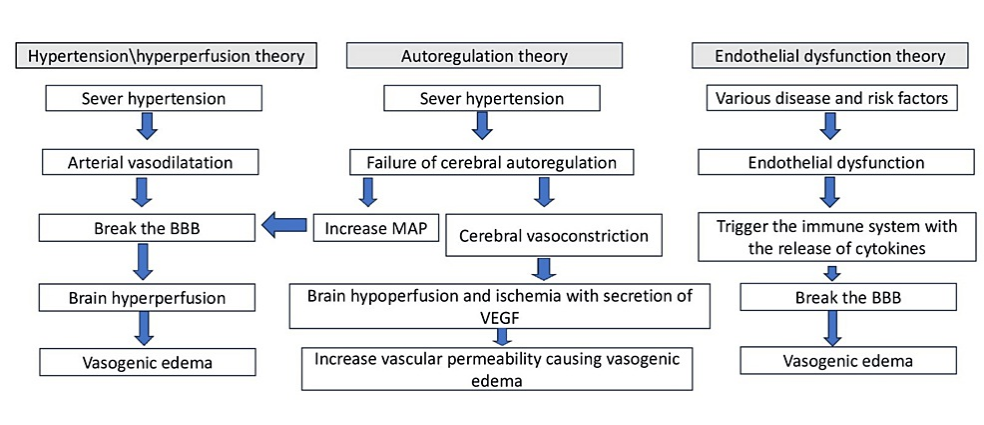
Pathophysiology of posterior reversible encephalopathy syndrome (PRES). BBB: blood-brain barrier; MAP: mean arterial pressure; VEGF: vascular endothelial growth factor The image is created by the authors of this study.

Despite ongoing research, the precise pathophysiology of PRES is complex and remains incompletely understood. Several mechanisms have been postulated to contribute to its development. Three main theories have been proposed to explain the mechanisms involved as follows: hypertension/hyperperfusion, autoregulation, and endothelial theories (Figure 1).

The hypertension/hyperperfusion theory postulates that elevated systemic blood pressure exceeding the threshold of cerebral autoregulation, typically associated with a rapid surge, triggers arteriolar dilation and subsequent brain hyperperfusion. This excessive blood flow compromises the integrity of the blood-brain barrier (BBB), accumulating fluid in the brain parenchyma and causing vasogenic edema [31].

The autoregulation theory suggests that hypertension in PRES patients disrupts the brain's ability to regulate its blood flow automatically. Normally, the brain maintains a balance of blood vessel dilation and constriction to ensure proper perfusion. However, when this process is interrupted by severe hypertension, the autoregulatory mechanism breaks down, leading to increased mean arterial pressure (MAP). This increase in MAP, exceeding the normal range (up to 160-200 mmHg), is believed to contribute to PRES symptoms. Importantly, the autoregulation threshold is lower in children (40 mmHg) compared to adults (50-60 mmHg). This explains why children with PRES often have lower MAPs than adults [31-34].

The autoregulation theory proposes that hypertension triggers the autoregulation system, causing brain vessel constriction, hypoperfusion, and ischemia. The resulting hypoxia leads to the secretion of vascular endothelial growth factor (VEGF) from cerebral vessels, which increases permeability and hence causes vasogenic edema [35,36].

PRES can occur even in the absence of hypertension, and 15-20% of patients with PRES have normal or only slightly high blood pressure [36,37]. The endothelial dysfunction hypothesis proposes that various disease entities induce endothelial dysfunction, triggering immune system activation and subsequent production of cytokines (inflammatory mediators). This disrupts the normal homeostasis of the blood-brain barrier (BBB), leading to fluid leakage and edema within the cerebrovascular endothelium.

Additionally, chemotherapeutic and immunosuppressive agents can directly impact the cerebrovascular endothelium, causing endothelial dysfunction with capillary leakage and resulting in vasogenic brain edema. Notably, chemotherapeutic-associated PRES can occur even in normotensive individuals [38]. However, in such pathological states with concomitant hypertension, vasoconstriction further exacerbates the inflammatory endothelial dysfunction. This synergistic effect ultimately results in hypoxia and subsequent vasogenic edema [39].

The specific mechanism underlying PRES in each case may depend on the condition's underlying cause. For instance, PRES associated with hypertension is more likely to involve the hyperperfusion theory. At the same time, PRES triggered by sepsis or other inflammatory conditions is more likely to involve the endothelial theory.

Effective treatment for PRES requires addressing the underlying cause and predisposing factors. In cases of hypertension-induced PRES, blood pressure control is paramount. Additionally, various medications may be employed to reduce cerebral edema and manage seizures.

Clinical features

PRES often has vague and non-specific symptoms that appear suddenly or gradually over hours or days but rarely worsen over weeks. The symptoms are non-specific, with focal or generalized seizures being the most common, observed in approximately 90% of cases, followed by visual disturbances, such as hemianopia, blurred vision, cortical blindness, or visual hallucinations, altered mental state, and headache [40,41]. Less common manifestations include hemiparesis, dysarthria/aphasia, ataxia, involuntary movements, dizziness/vertigo, and other focal neurological signs [15,25,26,41].

Neuroimaging features of PRES

Neuroimaging, including CT scans and MRIs, plays a crucial role in ruling out other possible diagnoses and confirming PRES [4,5]. While CT scans are often the first imaging modality used to assess potential PRES, especially for patients with sudden changes in mental status or seizures, it is essential to note that they are not always reliable for confirming the diagnosis [9]. They are also valuable in situations where MRI technology is not readily accessible. Early brain imaging in children with PRES typically reveals cortical and subcortical vasogenic edema in a symmetrical pattern of abnormalities in the parietal-occipital region [42]. These abnormalities are most commonly detected within the first 24 hours of symptom onset. MRI is significantly more sensitive for detecting these abnormalities than computed tomography (CT) scans, with 98% and 46% detection rates, respectively [3,11]. Both clinical symptoms and suggestive radiological findings are necessary for a definitive PRES diagnosis. Though the characteristic radiological patterns of PRES in children resemble those of adults, children tend to present with atypical features more often.

**Figure 2:**
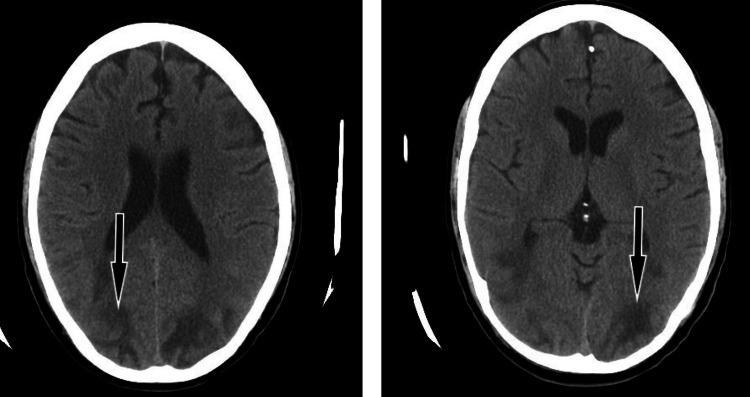
Axial non-enhanced CT scan of the brain. There are areas of ill-defined hypoattenuation involving predominantly the subcortical white matter in both parietal and occipital lobes (arrows). There is no acute intracranial hemorrhage. All images are the original work of the author.

Brain CT scans can reveal specific changes in PRES patients, including low-density areas appearing in symmetrical patterns across both hemispheres, particularly in areas surrounding large blood vessels, along the superior frontal sulcus, and predominantly in the parietal and occipital lobes (Figure 2) [43].

Despite the ability of brain CT scans to definitively diagnose PRES, their sensitivity is surpassed by MRI [3,43]. Patients with highly suggestive clinical symptoms of PRES but negative CT findings should undergo an MRI when their clinical status becomes stable [44].

**Figure 3:**
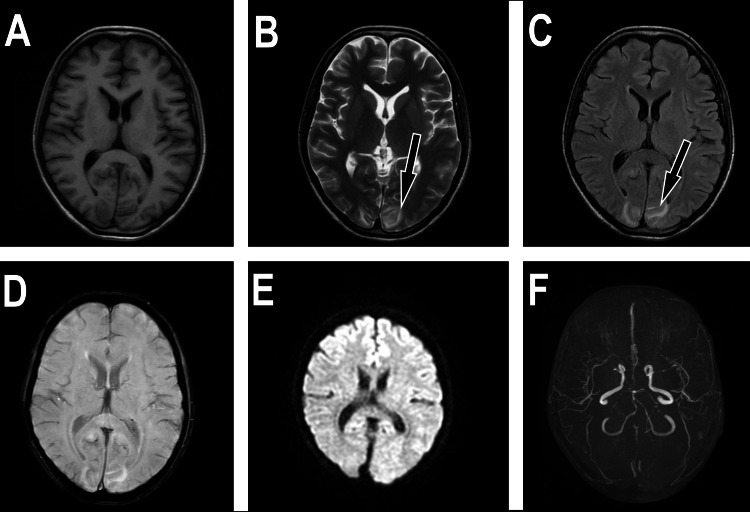
Axial MRI images of brain without contrast. The study demonstrated cortical and subcortical abnormal hypointensity on the T1-weighted image (A), hyperintense signal intensity on the T2-weighted image (B), and fluid-attenuated inversion recovery (FLAIR) (C) (arrows on B and C). There is no evidence of intraparenchymal hemorrhage on the susceptibility-weighted image (SWI) (D) or restricted diffusion on diffusion-weighted imaging (E). Time of flight MR angiography (TOF MRA) showed vascular irregularity. All images are the original work of the author.

MR imaging offers higher sensitivity than CT scanning for detecting vasogenic edema [3]. This edema appears hyperintense on T2-weighted and FLAIR sequences and hypointense on T1-weighted images. Additionally, it exhibits an increased apparent diffusion coefficient (ADC) with normal diffusion-weighted imaging (DWI) (Figures 3A-3F) [1,3,14,33,45].

Like the findings in brain CT scans, four primary descriptive variations are observed in approximately 70% of patients. These four types of vasogenic edema patterns have been identified based on their location, including classic parieto-occipital pattern involving near the border zone between the middle cerebral artery (MCA) and the posterior cerebral artery (PCA), affecting the parietal and occipital lobes, superior frontal sulcus pattern predominantly affects the area along the watershed zone between the anterior cerebral artery (ACA) and the MCA, particularly around the superior frontal sulcus, holohemispheric watershed pattern affects a large area of both hemispheres, encompassing the ACA-MCA watershed zone, and central pattern which occurs less frequently and is located deep within the brain, involving the deep white matter, basal ganglia, thalami, and brainstem [3,26,33].

Atypical MRI lesions are significantly more prevalent in children with PRES than in adults, with reported incidence rates ranging from 61% to 82% in children compared to 10% to 58% in adults [46]. This observation is supported by evidence from several studies. Additionally, the involvement of the superior frontal sulcus on MRI is also significantly more frequent in children with PRES compared to adults, as demonstrated in studies.

Atypical MRI findings show the involvement of gray matter, the frontal lobes, the thalamus, the brainstem, and the cerebellum [42]. Atypical findings also include unilateral/asymmetric involvement, restricted diffusion, intracerebral hemorrhage, microhemorrhages, and contrast enhancement, which are considered uncommon [47]. Lesions may be asymmetric in about 50% of cases and unilateral on rare occasions [37,48].

Atypical MRI findings can suggest cytotoxic edema caused by acute ischemia, which leads to restricted diffusion manifested as reduced ADC and increased DWI signal [49]. Furthermore, children with PRES exhibit restricted diffusion on MRI with a higher frequency (15-42%) compared to adults (15-30%), as reported. Although abnormal contrast enhancement is visible on T1-weighted scans after gadolinium injection in 37-43% of cases, possibly due to blood-brain barrier disruption, it does not seem to significantly affect patient prognosis [16].

Intracranial hemorrhage emerges as a frequent complication of pediatric PRES, with prevalence ranging from 8% to 28% [10,25,49], while adult series report rates between 10% and 25% [33]. Hemorrhage in PRES is believed to potentially arise from the rupture of small pial vessels in situations of extreme hypertension or during reperfusion after a period of vasoconstriction [16]. The hemorrhages manifest in various forms, including punctuate microhemorrhages, subarachnoid hemorrhage, and lobar hematoma [50,51]. Among these, punctuate microhemorrhages are the most common, but their clinical significance remains uncertain [33]. However, more widespread parenchyma hematoma can potentially lead to long-term consequences that worsen the prognosis [2].

Compared to CT scans and conventional T2-weighted Gradient Echo image (T2 GRE), susceptibility-weighted imaging (SWI) demonstrates increased sensitivity for detecting cerebral hemorrhages in PRES. This enhanced sensitivity stems from SWI's ability to visualize both frank intraparenchymal hemorrhages and early microhemorrhages associated with PRES. Moreover, recent studies utilizing SWI have identified a significantly higher prevalence of hemorrhage in PRES, with rates ranging from 26% to 64% [18,52].

Additionally, advanced neuroimaging techniques, including computed tomography angiography (CTA), magnetic resonance angiography (MRA), and conventional digital subtraction angiography (DSA), can potentially reveal vascular abnormalities in patients with PRES (Figure 3F). These abnormalities may include vasospasm, arteritis, diffuse or focal vasoconstriction, vasodilatation, and the characteristic "string-of-beads" appearance [33,43].

Magnetic resonance spectroscopy (MRS) provides valuable insights into brain chemicals, cell activity, and membrane integrity. This information can help diagnose PRES, predict its outcome, and understand its cause [53-55]. Most PRES cases show reduced ratios of N-acetyl aspartate/creatine ratio (NAA/Cr) and N-acetyl aspartate/choline ratio (NAA/Chol), indicating potential changes in neuronal function and metabolism [53-57]. Despite normalization of structural MRI and clinical resolution, NAA remains low, and Chol remains high in the subacute phase of the PRES [53,54,58].

Research findings on lactate (Lac) levels in PRES are inconsistent. Kwon et al. reported four pediatric PRES cases with no changes in NAA, Chol, or Cr but elevated Lac levels. In follow-up MRS examinations, nearly all cases showed complete recovery [25]. The presence of Lac raises concerns of tissue damage, potentially permanent, due to an infarct or another underlying pathology [59].

The results of CT and MRI perfusion studies in PRES show discrepancies. Some investigations demonstrate elevated perfusion, marked by increased cerebral blood flow (CBF), cerebral blood volume (CBV), and reduced time to peak (TTP) and mean transit time (MTT) [60-65]. Conversely, other studies present conflicting outcomes, indicating vasoconstriction, reduced CBF, almost normal CBV, and heightened values for TTP, MTT, and time to drain (TTD) [66-71]. The contradictory findings in perfusion studies likely mirror the intricate pathophysiology of PRES and the dynamic vascular alterations that occur throughout the course of the disease.

Despite being a clinical diagnosis potentially confirmed by brain imaging, PRES can occur without any abnormalities in neuroimaging [72]. Fugate and Rabinstein suggest that a patient experiencing at least one acute neurological symptom (seizures, confusion, headache, or visual disturbances) alongside at least one risk factor (severe hypertension, blood pressure fluctuations, kidney failure, immunosuppressant use, chemotherapy, eclampsia, or an autoimmune disorder) should raise suspicion for PRES clinically, regardless of the MRI results [33]. Permanent neurological damage is a potential consequence of PRES, affecting as many as 12% of patients. Recurrence and permanent neurological sequelae are additional concerns, and unfortunately, PRES also has a mortality rate of 16% [73,74].

Differential diagnosis

The differential diagnosis of pediatric PRES presents a significant challenge due to the broad spectrum of clinical presentations and its overlap with other acute neurological disorders. The differentials include infectious/autoimmune encephalitis and acute disseminated encephalomyelitis (ADEM). Vascular diseases such as ischemic stroke, including watershed infarction, top of basilar artery syndrome, and cerebral vein thrombosis, are essential considerations. Conditions such as mitochondrial myopathy encephalopathy lactic acidosis, stroke-like episodes syndrome (MELAS), post-transplant lymphoproliferative disease, ictal or post-ictal state, central nervous system vasculitis, osmotic demyelination syndrome, and toxic leukoencephalopathy are important differential diagnoses [2,4,33].

Accurate diagnosis of pediatric PRES necessitates careful assessment of clinical history, meticulous neuroimaging analysis, and selective laboratory investigations [4]. Moreover, the non-specific clinical presentation emphasizes the critical role of characteristic MRI findings in excluding alternative diagnoses.

## Conclusions

PRES is a complex and heterogeneous clinical-radiological entity characterized by acute neurological symptoms, including seizures and reversible parieto-occipital vasogenic edema on MRI. The underlying pathophysiology of PRES is unknown. The suggested cerebral endotheliopathy leads to BBB disruption and subsequent vasogenic edema. PRES is typically associated with specific predisposing conditions, such as abrupt hypertension, renal disorders, immunosuppressant therapy, and organ transplantation. MRI remains the cornerstone for diagnosing PRES; however, established diagnostic criteria are still lacking. PRES typically resolves within days to weeks with prompt recognition and management of the underlying cause. Nonetheless, neurological sequelae and even mortality can occur, particularly in patients with intracranial hemorrhages.

Despite significant advancements in understanding and managing PRES, several critical aspects still need to be discovered. Further research is warranted to elucidate the underlying pathophysiological mechanisms, paving the way for targeted therapeutic strategies and improved clinical outcomes. From a clinical standpoint, the development of validated diagnostic tools and algorithms and the evaluation of potential treatment options should be prioritized through extensive, multicenter prospective studies.
